# Hepatoprotective Effect of a New FFAR1 Agonist—N-Alkylated Isobornylamine

**DOI:** 10.3390/molecules28010396

**Published:** 2023-01-03

**Authors:** Darya Pon`kina, Sergey Kuranov, Mikhail Khvostov, Nataliya Zhukova, Yulia Meshkova, Mariya Marenina, Olga Luzina, Tatyana Tolstikova, Nariman Salakhutdinov

**Affiliations:** N. N. Vorozhtsov Novosibirsk Institute of Organic Chemistry, Siberian Branch of the Russian Academy of Sciences, 9, Akademika Lavrentieva Ave., Novosibirsk 630090, Russia

**Keywords:** hepatoprotective effect, bornyl derivatives, FFAR1 agonist, carbon tetrachloride, liver injury

## Abstract

Free fatty acid receptor-1 (FFAR1) is one of the possible therapeutic targets in the search for new hepatoprotective drugs. FFAR1 agonists were found to have hypolipidemic, antifibrotic, anti-inflammatory, antiproliferative and antioxidant effects in addition to hypoglycemic action. In this work, we conducted a study of the hepatoprotective effect of the compound QS-528 (previously discovered as an agonist of FFAR1) at doses of 60, 90, 120 and 150 mg/kg on carbon tetrachloride (CCl_4_)-induced liver injury. At the end of the experiment, a biochemical blood assay demonstrated that the introduction of QS-528 dose-dependently reduces the levels of liver enzymes (AST, ALT and ALKP). Histological and morphometric studies of animals’ livers treated with QS-528 at doses of 120 and 150 mg/kg showed a decrease in degenerative/necrotic changes in hepatocytes and an increase in the regenerative activity of the liver. In addition, no toxicity at a single oral dose of 1000 mg/kg and an increase in HepG2 cell viability in vitro were found. Thus, the compound QS-528 was found to exhibit a hepatoprotective effect against CCl_4_-induced toxic liver damage.

## 1. Introduction

The liver is one of the most important human organs necessary to maintain the vital activity of the organism. It performs many functions in the regulation of body homeostasis. For example, it takes an active part in carbohydrate, lipid, amino acid and protein metabolism and also detoxifies and eliminates foreign substances from the body [1]. Hepatocyte damage by chemicals (drugs, ethanol, carbon tetrachloride, etc.) and infectious agents disrupts the normal functioning of the liver, leading to the development of various diseases, such as hepatitis, cirrhosis, liver failure, etc. [2]. In the period 2007–2017, the age-standardized prevalence of cirrhosis and chronic liver diseases increased by 10.4% with 1.5 billion cases in 2017 [3].

Therefore, in order to prevent the development and progression of liver diseases, it is necessary to protect it from the harmful effects of hepatotoxins and increase the detoxification function and resistance to pathological influences. Drugs that perform these functions are called hepatoprotectors [4]. To date, there are no hepatoprotective drugs that fully correspond to these requirements. In addition, some of these drugs cause adverse effects [5]. As a result, the search for new, more effective and less toxic hepatoprotective agents is still an urgent task for modern pharmacology.

One of the possible therapeutic targets of the search for new hepatoprotective drugs is free fatty acid receptor-1 (FFAR1) [6]. It is highly expressed in pancreatic B-cells, promoting insulin secretion in a glucose-dependent manner, and in enteroendorenal K and L intestinal cells, mediating glucose-dependent insulinotropic polypeptide (GIP) and glucagon-like peptide (GLP) secretion, respectively [7]. Although evidence for endogenous hepatic protein expression is lacking, FFAR1 mRNA has been detected and studied in hepatocytes and in the HepG2 cell line [8]. The exact role of FFAR1 in the regulation of liver function remains to be elucidated [9]. However, recent studies have shown that FFAR1 has a protective function in the liver: it decreases the levels of free fatty acids and triglycerides and reduces oxidative stress, fibrosis and inflammation [8,9]. A similar effect was found in the full FFAR1 agonist—SCO-267. The oral administration of this compound to mice with NAFLD has been shown to decrease liver mass, triglyceride and collagen production, and blood levels of alanine aminotransferase, without affecting food intake or blood glucose levels [10]. A study of another FFAR1 agonist, PBI-4050, showed a significant antifibrotic effect in mice with liver fibrosis induced by CCl_4_ [11]. On S. et al. found that docosahexaenoic acid (DHA), an endogenous FFAR1 agonist (and other GPC receptors), mediates its antilipogenic effect via FFAR1 in hepatocytes [12]. The increased expression of these receptors in mouse hepatocytes under the influence of DHA was revealed, and their key role in the inhibition of lipid accumulation in cells was shown via the reduced expression of lipogenic enzymes. Thus, FFAR1 agonists may have a hepatoprotective effect either directly on hepatocytes or indirectly through the glucose-dependent insulinotropic pathway. Given the above, we can conclude that a very promising direction of pharmacology is the search for new hepatoprotective compounds among FFAR1 agonists.

**Figure 1:**
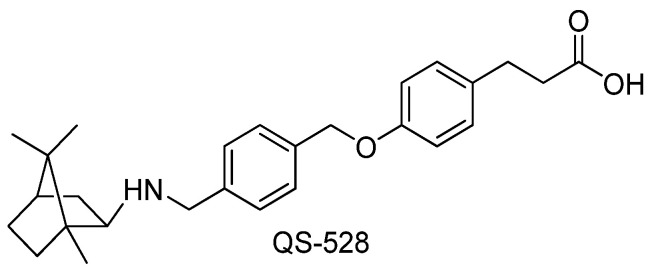
N-alkyl derivative of isobornylamine.

Previously, we have demonstrated that the compound QS-528 (Figure 1) at a dose of 30 mg/kg, which was discovered as an FFAR1 agonist [13], exhibited a hepatoprotective effect in obese mice with type 2 diabetes (C57Bl/6^Ay^ mice). It resolved liver fatty degeneration, which improved their glucose tolerance [13,14]. In the present work, we investigated QS-528 for its hepatoprotective effect on CCl_4_-induced liver hepatotoxicity, which is the standard model for studying this effect [15]. In addition, we examined the ability of QS-528 to directly affect liver cells by evaluating cell viability and glucose consumption in a HepG2 cell line.

## 2. Results

### 2.1. Biochemical Blood Assay

A biochemical study showed that the introduction of QS-528 dose-dependently reduced the concentration of ALT in mouse blood, and a more pronounced effect was observed at doses of 120 and 150 mg/kg (Table 1). A decrease in the concentration of AST was also noted after the introduction of QS-528 at a dose of 120 mg/kg, similar to the mice in the positive control group (Table 1). In addition, a trend toward a decrease in the concentration of ALKP was observed. The concentration of TP was determined in order to assess the synthetic liver function. It was the same in all studied groups.

### 2.2. Histological Examination

**Figure 2:**
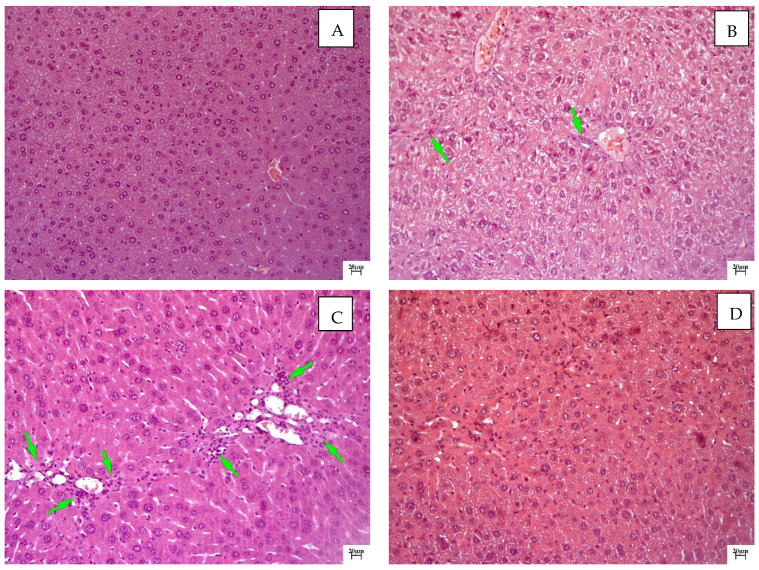
Histological examination of mouse livers after 3 weeks of the experiment: (**A**) Healthy control. In hepatocytes—small vesicular lipid infiltration and heterogeneity of the hepatocyte population: differences in the size of cells and nuclei. Staining with hematoxylin–eosin, ×200. (**B**) Mice treated with CCl_4_ alone. Hydropic degeneration and necrosis of hepatocytes. Green arrows indicate necrosis of hepatocytes. Staining with hematoxylin–eosin, ×200. (**C**) Mice treated with CCl_4_ alone. Lymphocyte-infiltrated periportal necrosis, fatty infiltration, and hepatocyte population heterogeneity: differences in cell and nuclear size. Green arrows indicate necrosis of hepatocytes. Staining with hematoxylin–eosin, ×200. (**D**) Mice treated with silymarin 100 mg/kg and CCl_4_. Small vesicular degeneration of hepatocytes, thin threads of connective tissue and absence of cellular and nuclear polymorphism of hepatocytes. Staining with hematoxylin–eosin, ×200.

In untreated mice (intact control), the liver architectonics were found to be preserved, and the bile capillaries, veins and arteries had a typical structure. Small vesicular lipid infiltration was observed in hepatocytes. The hepatocyte population was characterized by slight heterogeneity in terms of the cell size and nuclei. Kupffer cells and mononuclear leukocytes were determined in sinusoids (Figure 2A).

Chronic toxic liver damage was detected in animals in the negative control group. The predominant type of dystrophy was small vesicular dystrophy (Figure 2B). Small-focal necrosis of hepatocytes with severe lymphocytic infiltration and with Kaunsilmen’s bodies in sinusoids were observed in the liver. An increase in the regenerative activity of hepatocytes (mitoses) was observed against the background of the lesions described above, which was manifested by pronounced nuclear and cellular polymorphism (Figure 2C). Regeneration in this group was incomplete, which was realized by the hypertrophy and hyperplasia of hepatocytes. Portal tracts were dilated and infiltrated with lymphocytes and macrophages. The total narrowing of the sinusoidal lumen with signs of the increased activity of Kupffer cells was noted against the background of pronounced venous plethora, which was accompanied by a violation of transsinusoidal metabolism and cholestasis.

Improvement in the pathological process was noted after the introduction of silymarin at a dose of 100 mg/kg. Moderately pronounced signs of toxic liver damage persisted in the form of minor vesicular fatty degeneration and the lesser focal necrosis of hepatocytes (Figure 2D). Fibrotic liver changes were observed in the form of thin strands, spreading mainly perisinusoidally. Connective tissue strands were infiltrated with macrophages and mononuclear leukocytes. A large number of activated Kupffer cells were found in sinusoids, which may indicate the retention of fibrotic processes. A more monomorphic character of the hepatocyte population was noted: signs of nuclear cell polymorphism were weakly expressed, and an increase in the number of hepatocytes was visually noted.

**Figure 3:**
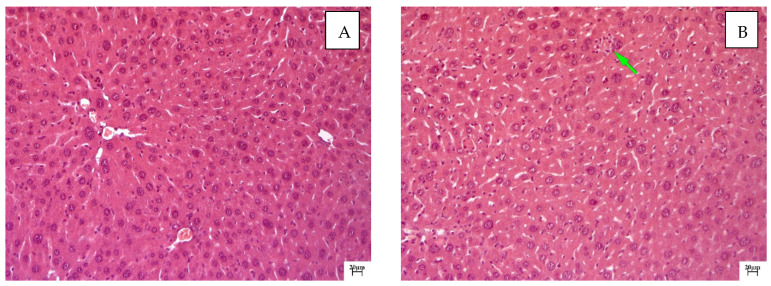
Histological examination of a mouse liver treated with QS-528 at doses of 60 and 90 mg/kg for 3 weeks: (**A**) Mice treated with QS-528 at a dose of 60 mg/kg. Severe hepatocyte hypertrophy and nuclear hyperplasia. Staining with hematoxylin–eosin, ×200. (**B**) Mice treated with QS-528 at a dose of 90 mg/kg. Cell necrosis with infiltration by lymphocytes and macrophages. Green arrow indicates necrosis of hepatocytes. Staining with hematoxylin–eosin, ×200.

QS-528 at doses of 60 and 90 mg/kg slightly reduced the severity of dystrophic changes in hepatocytes. Just like in the negative control group, small vesicular lipid degeneration was observed in hepatocytes. The polymorphism of hepatocytes due to increased regenerative activity was pronounced (Figure 3A). Reparative regeneration in this case was incomplete and was realized by the mechanism of compensatory hyperplasia and hypertrophy. Cell necrosis with infiltration by lymphocytes and macrophages was noted in the periportal zones (Figure 3B). Sinusoids were narrowed, and Kupffer cells and monocytes were determined in their lumens. The introduction of the drug at this dose did not have a pronounced effect on the development degree of fibrotic changes.

**Figure 4:**
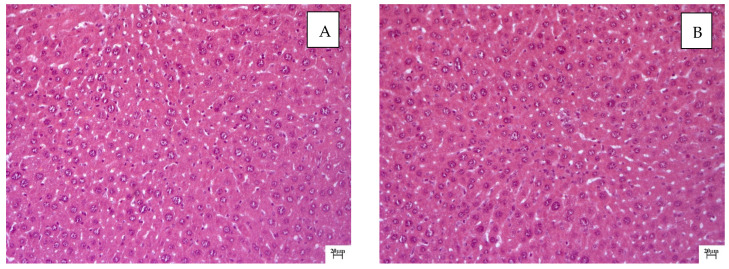
Histological examination of mouse livers treated with QS-528 at doses of 120 and 150 mg/kg for 3 weeks: (**A**) Mice treated with QS-528 at a dose of 120 mg/kg. The absence of heterogeneity of hepatocytes and fatty degeneration indicates the normalization of reparative processes. Staining with hematoxylin–eosin, ×200. (**B**) Mice treated with QS-528 at a dose of 150 mg/kg. Improvement in the structure of hepatocytes, lack of heterogeneity and dystrophy. Staining with hematoxylin–eosin, ×200.

QS-528 at doses of 120 and 150 mg/kg, in contrast to animals treated with this substance at doses of 60 and 90 mg/kg, had a positive effect on reparative processes in the liver. The heterogeneity of hepatocytes was manifested in the form of a slight disturbance of the nuclei. Small vesicular lipid degeneration was recorded in individual animals and was focal in nature (Figure 4A,B). Large cells (macrophages) were observed periportally. Kupffer cells and lymphocytes were detected in the sinusoids. Reparative regeneration in this case was realized by the mechanism of compensatory hyperplasia, which is typical of polyploidy.

Based on the histological examination data, it can be concluded that QS-528 at doses of 120 and 150 mg/kg had a pronounced positive effect on the course of reparative processes in the livers of mice.

### 2.3. Relative Density of Nuclei and Cytoplasm of Hepatocytes, Sinusoids and Liver Necrosis

The relative volume of the nuclei and cytoplasm of hepatocytes, as well as the volume of sinusoids and hepatic necrosis, was calculated to quantify the ability of QS-528 to restore the state of the mouse liver after exposure to CCl_4_. The greater the volume density of the nuclei and cytoplasm of hepatocytes and sinusoids and the lower the density of liver necrosis, the better the hepatoprotective effect of the test compound.

**Figure 5:**
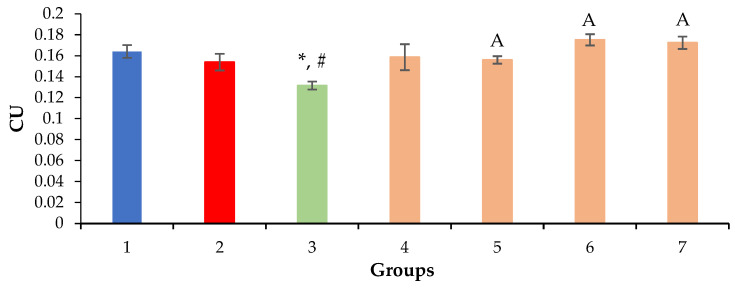
Volume density of hepatocyte nuclei. * *p* ≤ 0.05 as compared to the negative control; ^#^ *p* ≤ 0.05 as compared to the intact control; ^A^ *p* ≤ 0.05 as compared to silymarin 100 mg/kg. CU: conventional units; 1: intact control; 2: negative control; 3: positive control; 4: mice treated with QS-528 at a dose of 60 mg/kg; 5: mice treated with QS-528 at a dose of 90 mg/kg; 6: mice treated with QS-528 at a dose of 120 mg/kg; 7: mice treated with QS-528 at a dose of 150 mg/kg.

**Figure 6:**
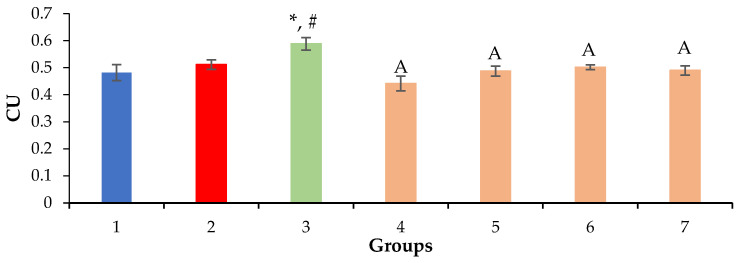
Volume density of hepatocyte cytoplasm. * *p* ≤ 0.05 as compared to the negative control; ^#^ *p ≤* 0.05 as compared to the intact control; ^A^ *p* ≤ 0.05 as compared to silymarin 100 mg/kg. CU: conventional units; 1: intact control; 2: negative control; 3: positive control; 4: mice treated with QS-528 at a dose of 60 mg/kg; 5: mice treated with QS-528 at a dose of 90 mg/kg; 6: mice treated with QS-528 at a dose of 120 mg/kg; 7: mice treated with QS-528 at a dose of 150 mg/kg.

Based on the results of the morphometric evaluation, the prolonged administration of a 0.5% CCl_4_ solution was found to have no effect on the volume density of the nuclei and cytoplasm of hepatocytes, except in mice in the positive control group (Figure 5 and Figure 6). With the introduction of silymarin, a decrease in the volume density of nuclei and an increase in the hepatocyte cytoplasm volume were observed in comparison with the intact and negative control groups (Figure 5 and Figure 6), which is probably due to the different regeneration modes of hepatocytes.

**Figure 7:**
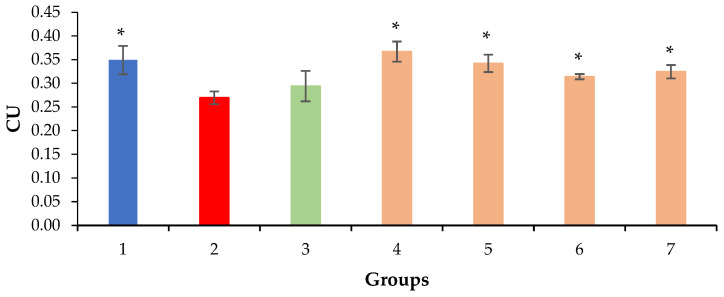
Volume density of liver sinusoids. * *p* ≤ 0.05 as compared to the negative control. CU: conventional units; 1: intact control; 2: negative control; 3: positive control; 4: mice treated with QS-528 at a dose of 60 mg/kg; 5: mice treated with QS-528 at a dose of 90 mg/kg; 6: mice treated with QS-528 at a dose of 120 mg/kg; 7: mice treated with QS-528 at a dose of 150 mg/kg.

The volume density of liver sinusoids after the administration of silymarin remained at the level of the negative control due to the preservation of the fibrotic process, which occurred mainly perisinusoidally (Figure 2 and Figure 7). An increase in the volume density of liver sinusoids, on the contrary, was observed in the experimental groups due to the resolution of small vesicular lipid degeneration and, as a result, a decrease in the compression of the sinusoidal lumen (Figure 3, Figure 4 and Figure 7).

**Figure 8:**
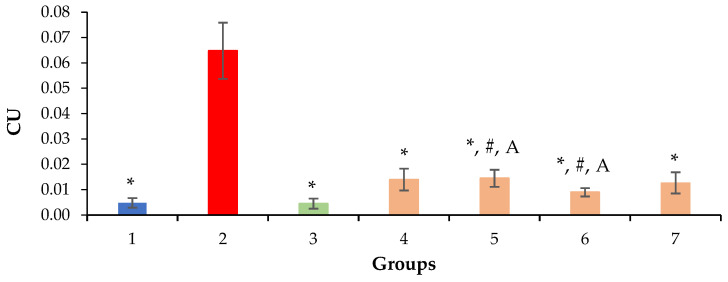
Volume density of liver necrosis. * *p* ≤ 0.05 as compared to the negative control; ^#^ *p* ≤ 0.05 as compared to the intact control; ^A^ *p* ≤ 0.05 as compared to silymarin 100 mg/kg. CU: conventional units; 1: intact control; 2: negative control; 3: positive control; 4: mice treated with QS-528 at a dose of 60 mg/kg; 5: mice treated with QS-528 at a dose of 90 mg/kg; 6: mice treated with QS-528 at a dose of 120 mg/kg; 7: mice treated with QS-528 at a dose of 150 mg/kg.

A decrease in the volume density of liver necrosis is one of the main indicators of the hepatoprotective effect, which can be observed with the introduction of silymarin at a dose of 100 mg/kg and QS-528 at all doses studied (Figure 8).

### 2.4. Acute Toxicity Study

**Figure 9:**
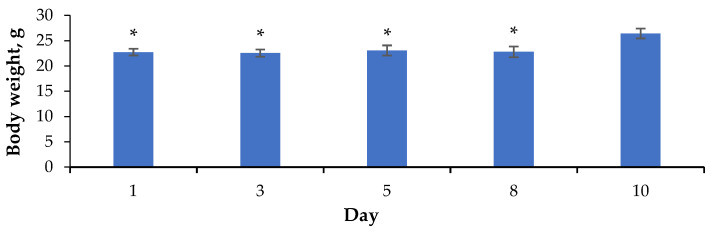
Body weight of CD-1 mice after a single injection of QS-528 at a dose of 1000 mg/kg. * *p* ≤ 0.05 as compared to the mouse weight after 10 days of QS-528 introduction.

According to the results of an acute toxicity study, QS-528 at a dose of 1000 mg/kg was found to have no adverse effect on the change in the body weight of mice during the 10 days of the study (Figure 9). Changes in the animals’ appearance and behavior were also not observed. No lethality was detected. Therefore, it may be concluded that QS-528 is a moderately toxic compound.

### 2.5. In Vitro Assay

In in vitro experiments, QS-528 at concentrations of 2.5 and 5 μM was found to slightly increase glucose consumption and lactate release in HepG2 cells but to significantly increase the number of cells. At higher concentrations (10–25 µM), QS-528 stimulates more glucose uptake by cells; however, their survival decreases. Metformin stimulates glucose consumption and lactate release in HepG2 cells at significantly higher concentrations (mM) without affecting cell numbers (Table 2).

## 3. Discussion

In this work, we examined the hepatoprotective effect of the compound QS-528 using a CCl_4_-induced model of hepatotoxicity. Liver injury caused by CCl_4_ is commonly used for the screening of hepatoprotective compounds [16]. CCl_4_ is a potent hepatotoxin capable of causing liver damage by activating free radicals, trichloromethyl (CCl_3_•) and trichloromethylperoxyl (CCl_3_OO•), mediated by cytochrome P450. Highly reactive radicals formed from CCl_4_ affect hepatocytes and cause structural and functional changes in their cell membranes, which in turn leads to the development of small vesicular dystrophy, inflammation, necrosis and fibrosis of the liver [17,18]. It is possible to simulate liver damage of varying severity depending on the dose, route and frequency of CCl_4_ administration [19,20,21]. Massive necrosis of hepatocytes can be achieved with this hepatotoxin [22], but it is very difficult to find protection against such a strong effect. By using a more gentle protocol for its administration, it is possible to simulate liver damage that will be closer in severity to that observed in metabolic disorders [23,24] or disorders associated with the intake of xenobiotics, such as drugs [25]. Based on this, we chose a gentle CCl_4_ introduction protocol. Carbon tetrachloride-induced damage to animals’ livers was significant but did not cause extensive necrosis of hepatocytes (Figure 2B,C and Figure 8). A moderate elevation of liver enzyme (ALT, AST and ALP) levels in the blood serum also indicated liver damage in animals treated with CCl_4_, because these enzymes enter the bloodstream after damaging hepatocytes [26] (Table 1).

The administration of QS-528 for three weeks to these mice produced a dose-dependent improvement in their liver condition. This could be observed in the biochemical (Table 1), histological (Figure 3A,B and Figure 4A,B) and morphometric results (Figure 8). The hepatoprotective effect was more pronounced at doses of 120 and 150 mg/kg. This confirmed an increase in the reparative regeneration of hepatocytes, a decrease in steatosis and necrosis of the liver (Figure 4A,B and Figure 8) and a decrease in ALT (Table 1). It is worth noting that there was a trend toward a decrease in ALKP activity at these doses, which suggests the stability of biliary dysfunction in the mouse liver during chronic hepatic injury with CCl_4_ [27]. The concentration of total protein remained at the level of the intact control after the administration of both CCl_4_ and the test compounds. This indicates the preservation of the synthetic liver function and is considered a contributory hepatoprotective mechanism that accelerates the regeneration process of liver cells [27]. A similar effect was observed in the positive control group (Figure 2D and Figure 8; Table 1); therefore, it can be argued that QS-528 at a dose of 120 or 150 mg/kg is not inferior to the reference hepatoprotector silymarin at a dose of 100 mg/kg.

Usually, the hepatoprotective drug efficacy is due to either restoring the normal hepatic physiology or reducing the harmful effect that has been caused by the hepatotoxic agent [2]. Previously, it was discovered in vitro that QS-528 activates FFAR1 and therefore is an agonist of this receptor [13]. For this reason, it can be assumed that QS-528, like the full FFAR1 agonist SCO-267, resolves fatty liver degeneration by reducing the level of oxidative stress caused by free radicals formed during CCl_4_ metabolism [10]. This mechanism seems to be universal for hepatoprotective compounds since the positive control silymarin enhances hepatic glutathione levels, which is commonly associated with the antioxidant defense of the liver [28]. In addition, it is possible that the hepatoprotective effect of QS-528 on this hepatotoxicity model is also associated with the ability of FFAR1 agonists to prevent the development of CCl_4_-induced fibrosis via the regulation of macrophages, fibroblasts/myofibroblasts and epithelial cells [11]. The hepatoprotective effect of this substance type is most often studied in the non-alcoholic fatty hepatosis model [29], which is a complication of type 2 diabetes [30]. QS-528, studied in this article, previously demonstrated a hepatoprotective effect in such a model (obese mice with type 2 diabetes—C57Bl/6Ay) [14]. A pronounced improvement in the condition of the animals’ livers was noted in that work due to a decrease in fatty hepatosis, which is typical of the animals of this line [31]. This effect helped to restore the insulin liver sensitivity of the liver and led to an increase in the glucose tolerance of mice. We have shown in this work that QS-528 can also have a hepatoprotective effect on toxic liver damage.

The direct effect of QS-528 on liver cells was also revealed in our in vitro experiments. Cell viability increased in HepG2 cells exposed to QS-528 at low concentrations. At the same time, the stimulation of glucose consumption by cells was also observed, but at higher concentrations. It is worth noting that the increase in cell viability is not a consequence of cells’ glucose consumption stimulation, since the addition of metformin, a stimulator of AMP-activated protein kinase a1 (AMPKa1) [32], did not lead to an increase in the number of cells.

An important fact is the lack of toxicity of this compound in vivo at a dose of 1000 mg/kg, which suggests that its LD50 is significantly higher than this dose. This, in turn, makes QS-528 safe for long-term administration, which is an extremely important characteristic of a potential drug [33].

Therefore, based on the data obtained, it can be concluded that QS-528 dose-dependently reduces the syndromes of cytolysis and cholestasis, resolves fatty liver degeneration and reduces the amount of necrosis in mice after CCl_4_ intoxication. It demonstrates no acute toxicity at a dose of 1000 mg/kg and stimulates HepG2 cell growth in vitro. Together, these results indicate that QS-528 exhibits a hepatoprotective effect; however, further studies are required to better understand its pharmacological action.

## 4. Materials and Methods

### 4.1. Investigated Compound

Compound QS-528 (Figure 1) was synthesized according to previously described procedures (see Supplementary Materials) [13] and used in the experiment at doses of 60, 90, 120 and 150 mg/kg as a hydrochloride salt.

### 4.2. Animals

Male CD-1 mice weighing 25–30 g obtained from the specific pathogen-free vivarium of the Institute of Cytology and Genetics of the Siberian Branch of the Russian Academy of Sciences were used in the experiment. The animals were kept under standard conditions with free access to water and food in humidity- and temperature-controlled rooms on a 12/12 h light-and-dark cycle. All manipulations with animals were carried out in strict accordance with the legislation of the Russian Federation, a decree of the Ministry of Health of the Russian Federation No. 199n of 4 January 2016 and the provisions of Directive 2010/63/EU of the European Parliament and of the Council of the European Union of 22 September 2010 on the protection of animals used for scientific purposes. The experiment was approved by the Ethics Committee of the N.N. Vorozhtsov Institute of Organic Chemistry SB RAS (protocol No. P-01-04-2022-14).

### 4.3. Carbon Tetrachloride (CCl_4_)-Induced Liver Hepatotoxicity

Mice were divided into 7 groups (9 animals per group). The animals were treated daily per os with the test compounds for 3 weeks. Group 1 “Intact control”: mice were injected with vehicle only (distilled water + 2 drops of Tween 80); group 2 “Negative control”: mice were injected with vehicle (distilled water +2 drops Tween 80) + 0.5% CCl_4_ solution in olive oil; group 3 “Positive control”: silymarin at a dose of 100 mg/kg (Legalon 70, MADAUS, GmbH) + 0.5% CCl_4_ solution in olive oil; group 4: QS-528 at a dose of 60 mg/kg + 0.5% CCl_4_ solution in olive oil; group 5: QS-528 at a dose of 90 mg/kg + 0.5% CCl_4_ solution in olive oil; group 6: QS-528 at a dose of 120 mg/kg + 0.5% CCl_4_ solution in olive oil; group 7: QS-528 at a dose of 150 mg/kg + 0.5% CCl_4_ solution in olive oil. Doses used were selected according to preliminary experiments (data not published) on the same animal model, in which QS-528 was studied at doses of 30 and 60 mg/kg. A slight hepatoprotective effect was observed only at a dose of 60 mg/kg. According to this, several doses in increments of 30 mg/kg were chosen for the present work.

Carbon tetrachloride (CCl_4_) solution was administered per os 2 times a week one hour after the administration of the test compound. At the same time, groups 2–7 received a 5% ethyl alcohol solution instead of water ad libitum throughout the experiment. At the end of the experiment (day 22), mice were decapitated, blood was taken for biochemical analysis and the liver was taken for histological examination.

### 4.4. Biochemical Assays

Blood was centrifuged at 1640 g for 15 min to obtain serum. Total protein (TP), alanine aminotransferase (ALT), aspartate aminotransferase (AST) and alkaline phosphatase (ALKP) were quantified in all groups using standard diagnostic kits (Vector-Best, Russia) and a Stat Fax 3300 spectrophotometer (USA).

### 4.5. Histological Liver Examination

The liver was fixed in 10% neutral buffered formalin for 7 days, then standard dehydration in ascending ethanol concentrations and xylene was carried out, and the specimens were embedded in paraffin on an AP 280 workstation using Histoplast with a melting point of 58 °C. Tissue slices with a thickness of 4.5 μm were prepared on a rotational NM 335E microtome with disposable interchangeable blades. The sections were stained with hematoxylin and eosin and examined under a light microscope at a magnification of ×100–200 to reveal histopathological changes, namely, fatty liver degeneration, necrosis, fibrosis, loss of cell boundaries, etc.

### 4.6. Morphometric Liver Analysis

Determination of the relative volume (volume density) of the hepatocyte nuclei and cytoplasm, sinusoids and liver necrosis was performed by calculating the number of dots (ends of test lines) hitting the profiles of the studied structures in the histological section. The volume density (Vv) of each structural liver component was calculated using the formula Vv = x:n, where x is the number of points per given component, and n is the total number of counted points.

### 4.7. Acute Toxicity Evaluation

In order to determine the acute toxicity (LD_50_), QS-528 was administered per os to CD-1 mice (n = 8) at a single dose of 1000 mg/kg. Animals’ behavior, body weight and lethality were evaluated over the next 10 days.

### 4.8. In Vitro Experiments

#### 4.8.1. Cell Culture

HepG2 cells were obtained from the shared research facility “Vertebrate cell culture collection” Institute of Cytology RAS. Cells were routinely cultured in high-glucose DMEM (Servicebio, Wuhan, China) containing 10% FBS (Sigma-Aldrich, São Paulo, Brazil), 100 μg/mL streptomycin, 100 U/mL penicillin and 0.25 μg/mL amphotericin B (Sigma-Aldrich, Saint Louis, MO, USA) at 37 °C in a 5% CO_2_ incubator (NuAire, Inc., Plymouth, MN, USA). Cells were passaged when cell fusion was over 80%, about twice a week.

#### 4.8.2. The Design of the Experiment on HepG2 Cells

Cells were seeded into 96-well plates (TPP, Trasadingen, Switzerland) at a density of 3 × 10^5^ cells/mL in high-glucose DMEM containing 10% FBS. After culturing for 24 h, the cells were washed with HBSS (Gibco, Paisley, Scotland, UK) twice, and the medium was replaced by serum-free low-glucose (5.5 mM glucose) DMEM (Servicebio, Wuhan, China) with QS-528 at various concentrations (2.5, 5 and 10 μM) and metformin (1, 2.5 and 5 mM) (CAS 1115-70-4 Acros Organics, Geel, Belgium). There were three to four replicate wells for each treatment. After 24 h, part of the supernatant was collected for glucose consumption and lactate release assay, while cell viability was measured using the MTT assay.

#### 4.8.3. Glucose Consumption and Lactate Release Assay

Glucose levels were assayed by a kit (Vector-Best, Novosibirsk, Russia) that was based on the glucose oxidase method using a photometer (Multiscan Ascent, Thermo Labsystems, Helsinki, Finland). Glucose consumption was calculated by subtracting the glucose concentration in the supernatant of the blank well (DMEM without cells) from the glucose concentration in the supernatant of the well with cells. Data are presented as the percentage of maximum glucose consumption (5.5 mM) and normalized to the control (treated with vehicle) for each plate. Meanwhile, the concentration of lactate in the supernatant was also determined by a commercial kit (Vector-Best, Novosibirsk, Russia). Lactate release is presented as the percentage of the maximum value (11 mM) and normalized to the control.

#### 4.8.4. MTT Assay for Cell Viability

A solution of MTT reagent (Thiazolyl Blue Tetrazolium Bromide (Panreac AppliChem, Darmstadt, Germany)) (5 mg/mL) was added to the medium in a 1:10 volume ratio and incubated for 4 h at 37 °C in a CO_2_ incubator. Then, the supernatant was removed, and the precipitate was dissolved with DMSO (Reagent Component, Moscow, Russia) for the determination of the optical density. Cell viability was calculated as percentage of the control group.

### 4.9. Statistical Analysis

Statistical analysis was performed in Statistica 7.0 software using one-way ANOVA, followed by the Fisher LSD test for multiple comparisons. All data are presented as mean ± SEM (standard error of the mean). Differences with *p* < 0.05 were considered statistically significant.

## 5. Conclusions

Based on the data obtained, it can be concluded that QS-528 dose-dependently reduces the syndromes of cytolysis and cholestasis, resolves fatty liver degeneration and reduces the amount of necrosis in mice after CCl_4_ intoxication. It demonstrates no acute toxicity at a dose of 1000 mg/kg and stimulates HepG2 cell growth in vitro. Together these results indicate that QS-528 exhibits a hepatoprotective effect; however, further studies are required to better understand its pharmacological action.

## Figures and Tables

**Table 1:** Blood biochemical parameters of CD-1 mice treated for 3 weeks with QS-528 at a dose of 60, 90, 120 or 150 mg/kg.

Group	ALT, U/L	AST, U/L	ALKP, U/L	TP, g/dL
Intact control	15.29 ± 1.28 *	41.49 ± 3.26 *	40.96 ± 5.63 *	85.30 ± 0.84
Negative control	26.5 ± 1.68	52.06 ± 2.56	68.01 ± 5.03	85.76 ± 0.74
Silymarin 100 mg/kg	20.75 ± 1.35 *	39.59 ± 2.71 *	43.08 ± 3.57 *	83.7 ± 1.00
QS-528 60 mg/kg	21.07 ± 1.95 *	44.48 ± 3.53	82.71 ± 14.25	84.23 ± 1.69
QS-528 90 mg/kg	25.82 ± 1.72	52.93 ± 4.41	75.62 ± 4.73	85.05 ± 1.43
QS-528 120 mg/kg	20.97 ± 1.39 *	40.83 ± 3.34 *	60.44 ± 6.02	84.98 ± 0.75
QS-528 150 mg/kg	14.95 ± 1.36 *^, #^	45.78 ± 6.93	54.35 ± 6.33	83.19 ± 1.18

* *p* < 0.05 as compared to the negative control; ^#^ *p* ≤ 0.05 as compared to silymarin 100 mg/kg. ALT: alanine aminotransferase; AST: aspartate aminotransferase; ALKP: alkaline phosphatase; TP: total protein.

**Table 2:** Effect of the QS-528 compound on the HepG2 cell line.

Group	Glucose Consumption, %	Lactate Release, %	Cell Viability, %
Control	24.98 ± 1.68	15.03 ± 0.59	100.00 ± 3.07
Metf. 1 mM	48.52 ± 2.87 ***	30.03 ± 3.04 ***	101.51 ± 1.90
Metf. 2.5 mM	62.32 ± 0.72 ***	46.08 ± 3.20 ***	91.11 ± 3.40
QS-528 2.5 μM	39.80 ± 2.75 **	25.58 ± 1.35 ***	146.31 ± 6.16 ***
QS-528 5 μM	37.79 ± 4.46 **	27.65 ± 1.15 ***	135.77 ± 4.19 ***
QS-528 10 μM	44.91 ± 3.00 ***	42.62 ± 1.20 ***	114.54 ± 3.95
QS-528 25 μM	85.52 ± 1.99 ***	61.61 ± 0.49 ***	109.57 ± 2.67

Data are represented as mean ± SEM. ** *p* < 0.01 and *** *p* < 0.001 as compared to control. Metf.—metformin.

## Data Availability

Not applicable.

## Supplementary Materials

The following supporting information can be downloaded at: https://www.mdpi.com/article/10.3390/molecules28010396/s1. Scheme S1. Synthesis of the target compound. Reagents and conditions: (a) NH2OH•HCl, AcONa•3H2O, MeOH, H2O; (b) NaBH4, NiCl2•6H2O, MeOH, −25 °C; (c) CH2(COOH)2, piperidine, pyridine, 65 °C; (d) H2SO4, MeOH, reflux; (e) NaBH4, NiCl2•6H2O, MeOH, 0 °C, (f) p-BrCH2C6H4CH2Br, K2CO3, acetone, reflux; (g) 1, DIPEA, CH3CN, reflux; (h) LiOH•H2O, MeOH/THF/H2O; (i) HCl, H2O.
