# Supplementary material for: Hepatoprotective Effect of a New FFAR1 Agonist—N-Alkylated Isobornylamine
Source: Molecules. 2023 Jan 3;28(1):396. doi: 10.3390/molecules28010396 (PMC9823450; doi:10.3390/molecules28010396)
Supplement: Supplementary file 1 [file molecules-28-00396-s001.zip › molecules-2128961-supplementary.pdf]

Supplementary Materials

Hepatoprotective Effect of a New FFAR1 Agonist—N-Alkylated Isobornylamine

Darya Pon`kina *, Sergey Kuranov, Mikhail Khvostov, Nataliya Zhukova, Yulia Meshkova, Mariya Marenina, Olga Luzina, Tatyana Tolstikova and Nariman Salakhutdinov

N. N. Vorozhtsov Novosibirsk Institute of Organic Chemistry, Siberian Branch of the Russian Academy of Sciences, 9, Akademika Lavrentieva Ave., Novosibirsk 630090, Russia

*Correspondence: daponkina@nioch.nsc.ru

Synthesis of QS-528

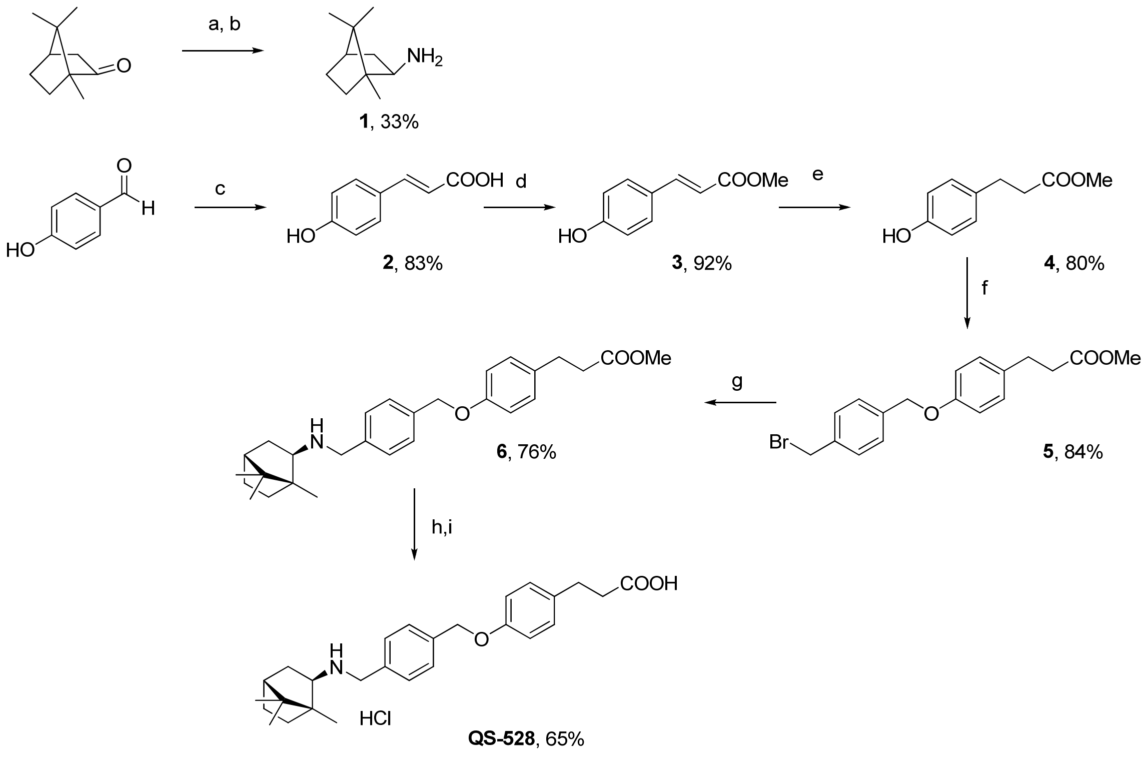

Scheme S1. Synthesis of the target compound. Reagents and conditions: (a) $NH_2OH•HCl$, $AcONa•3H_2O$, MeOH, H_2O; (b) $NaBH_4$, $NiCl_2•6H_2O$, MeOH, -25°C; (c) $CH_2(COOH)_2$, piperidine, pyridine, 65°C; (d) H_2SO_4, MeOH, reflux; (e) $NaBH_4$, $NiCl_2•6H_2O$, MeOH, 0°C, (f) p-$BrCH_2C_6H_4CH_2Br$, K_2CO_3, acetone, reflux; (g) 1, DIPEA, CH_3CN, reflux; (h) $LiOH•H_2O$, MeOH/THF/H_2O; (i) HCl, H_2O.

Optical rotation: polar 3005 spectrometer; $CHCl_3$ soln. ^1H and ^{13}C NMR spectra: Bruker spectrometers AV-400 at 400.13 MHz (^1H) and 100.61 MHz (^{13}C), AV-600 at 600.30 MHz (^1H) and 150.95 MHz (^{13}C) in $CDCl_3$; chemical shifts δin ppm relative to residual $CHCl_3$[δ($CHCl_3$)

7.26, δ($CHCl_3$) 77.00 ppm], J in Hz. The structure of the products was determined by analysing1H and 13C NMR spectra. HR-MS: DFS Thermo Scientific spectrometer in a full scan mode (15–500 m/z, 70eV electron impact ionisation, direct sample administration). Column chromatography was performed on silica gel (60–200 l, Macherey-Nagel. The purity of target compounds was determined by gas chromatography. All the target compounds reported in this paper have the purity of at least 95%. Spectral and analytical studies were carried out at the Collective Chemical Service Center of the Siberian Branch of the Russian Academy of Sciences. All chemicals were analytically pure and were used as received without purification prior to use. (+)-Camphor (>98.0%) was obtain from TCI (Product Number C0010). Dichloromethane was distilled over phosphorus pentoxide before use.

(1R,2R,4R)-1,7,7-trimethylbicyclo[2.2.1]heptan-2-amine 1

A mixture of (+)-camphor (13 mmol, 1.98 g), hydroxylamine hydrochloride (215 mmol, 1.77 g), and water (7 ml) was heated to 80°C, and methanol (7 ml) was added to dissolve the camphor. A solution of sodium acetate trihydrate (3.3 mmol, 4.5 g) in water (4 ml) was added, and the reaction mixture was heated under reflux at 100°C for 12 h. Upon removal of the methanol under reduced pressure, the white solid that precipitated was collected by filtration, washed with water (3x10 ml), and dried in vacuo to afford 1.95 g (90% yields) camphor oxime. The sodium borohydride (70.0 mmol , 2.66 g) was added portionwise to a solution of (1*S*)-camphor oxime (12 mmol, 1.95 g) and nickel dichloride hexahydrate (23 mmol, 5.54 g) in anhydrous methanol (40 ml) at –25°C over a period of 3 h. After completion of the addition, the resulting black slurry was stirred at this temperature overnight. The reaction mixture was then warmed to room temperature, and 25% ammonia solution (15 ml) in water (20 ml) was added with vigorous stirring. The resulting slurry was extracted with diethyl ether (3x70 ml), and the combined organic layers were washed with brine (20 ml), dried with anhydrous magnesium sulfate, filtered, and concentrated in vacuo. The crude product was purified by column chromatography (dichloromethane-methanol 100:0–90:10). Foamy white solid, 33% yield. ^{1}H NMR (400 MHz, $CDCl_3$) δ 0.81 (s, 3H), 0.86 (s, 3H), 0.96 (s, 3H), 0.97 - 1.08 (m, 2H), 1.21 - 1.28 (m, 2H), 1.48 - 1.57 (m, 2H), 1.65 - 1.77 (m, 3H), 2.69 (dd, *J* = 8.9 Hz and *J* = 5.1 Hz, 1H).

(E)-3-(4-hydroxyphenyl)acrylic acid 2

p-Hydroxybenzaldehyde (1.512 g, 12.3 mmol) and malonic acid (2.83 g, 27.2 mmol) were placed in a 10 ml flask and dissolved in 6.8 ml of pyridine. Piperidine (0.115 ml, 1.2 mmol) was added, and the reaction was placed in pre-heated to 72°C bath for 5 days (controlled by tlc CH_2Cl_2-ethyl acetate 4:1). Then, the mixture was transferred to a beaker, and 100 ml of water was added. Concentrated HCl was added drop-wise until pH 2-3. A white precipitate was collected using a Buchner funnel and dried under vacuum desiccator over P_2O_5. White powder, 83% yield. ^{1}H NMR

(300 MHz, DMSO-d_6) δ 6.27 (d, *J* = 15.9 Hz, 1H), 6.78 (d, *J* = 8.6 Hz, 2H), 7.40 - 7.56 (m, 3H), 9.84 - 10.27 (br.s, 1H)

(E)-methyl 3-(4-hydroxyphenyl)acrylate 3

(E)-3-(4-hydroxyphenyl)acrylic acid **2** (1.06 g 6.46 mmol) was dissolved in dry methanol. 2-3 drops of conc. sulfuric acid were added and solution obtained was refluxed for a two days until disappearing of starting material (controlled by tlc CH_2Cl_2-acetic acid 100:1). After completion of the reaction methanol was evaporated under vacuum. A residue was re-dissolved in ethyl acetate (30 ml) and washed with water (10 ml), brine (10 ml) and dried over magnesium sulfate. Solvent was evaporated and the residue was used in following step without further purification. White-off powder, 92% yield. ^{1}H NMR (400 MHz, $CDCl_3$) δ 3.81 (s, 3H), 6.30 (d, *J* = 16.0 Hz, 1H), 6.53 (br.s, 1H), 6.82 - 6.91 (m, 2H), 7.41 (d, *J* = 8.6 Hz, 2H), 7.64 (d, *J* = 16.0 Hz, 1H).

Methyl 3-(4-hydroxyphenyl)propanoate 4

To an ice-bath cooled solution of (E)-methyl 3-(4-hydroxyphenyl)acrylate **3** (0.845 g, 5.1 mmol) and nickel dichloride hexahydrate (0.12 g, 0.5 mmol) in dry methanol (10 ml) a sodium borohydride (0.5 g, 13.2 mmol) was added in small portion. After completion of reaction (controlled by GC-MS) 2.5 ml of saturated ammonium chloride solution was added and methanol was evaporated under reduced pressure. Water (5 ml) and aqueous ammonia (10ml) were added to the residue, and the product was extracted with ethyl acetate (3x15 ml). The extracts were combined, dried over Na_2SO_4, and evaporated. The crude product was purified by column chromatography (hexane-ethyl acetate 7:3). White solid, 0.67 g 80%. ^{1}H NMR (500 MHz, $CDCl_3$) δ 2.60 (t, *J* = 7.70 Hz, 2H), 2.88 (t, *J* = 7.70 Hz, 2H), 3.67 (s, 3H), 5.55 (br.s, 1H), 6.70 - 6.80 (m, 1H), 7.00 - 7.09 (m, 1H).

Methyl 3-(4-(4-(bromomethyl)benzyloxy)phenyl)propanoate 5

A solution of methyl 3-(4-hydroxyphenyl)propanoate **4** (1.15 g, 6.4 mmol) and potassium carbonate (2.55 g, 18 mmol) in acetonitrile (20 ml) was stirred for 1 hour at reflux. Then stirring was stopped, the reaction mixture was allowed to cool down and a precipitate was allowed to settle. The solution was decanted into an addition funnel with additional acetonitrile (40 ml). A solution of p-dibromoxylene (4.80 g, 18.2 mmol) in acetonitrile (20 ml) was charged to the same reaction flask. Then the solution of methyl 3-(4-hydroxyphenyl)propanoate **4** was slowly added to the reaction flask under reflux for 4 hours. The mixture obtained was refluxed for additional 2 hours and then was cooled down. A precipitate was filtered of and washed with chloroform (3x30ml). Combined organic filtrate was evaporated and part of the unreacted p-dibromoxylene was recrystallized from acetone. The residue was purified by column chromatography with dry loading (hexane-ethyl acetate 20:1 to 10:1 after separation of p-dibromoxylene). White solid, 81% yield.^{1}H

NMR (400 MHz, $CDCl_3$) δ 2.60 (t, *J* = 7.8 Hz, 2H), 2.89 (t, *J* = 7.8 Hz, 2H), 3.67 (s, 3H), 4.50 (s, 2H), 5.03 (s, 2H), 6.89 (d, J=8.7 Hz, 2H), 7.12 (d, *J* = 8.6 Hz, 2H), 7.41 (s, 4H).

Methyl 3-(4-(4-(((1R,2R,4R)-1,7,7-trimethylbicyclo[2.2.1]heptan-2-ylamino)methyl)benzyloxy)phenyl)propanoate 6

A solution of bromide **5** (1.18 mmol), amine (1.46 mmol) and N,N-diisopropylethylamine (2.01 mmol) in acetonitrile (7 ml) was refluxed for 2 hours (Control by tlc). Ethyl acetate (20 ml) and 5% sodium hydroxide (10 ml) solution were added. Organic layer was separated and water layer was extracted with ethyl acetate (2x10 ml). Combined organic layers were washed with water (10 ml), brine (10 ml) and dried over magnesium sulfate. Solvent was evaporated under vacuum and residue was purified over silica gel column chromatography using chloroform-methanol 100:1. White solid 76% yield. M.p. 89.7-90.0°C. $[\alpha]_D^{24}$ -46 (c 0.140, $CHCl_3$). IR (KBr,) ν/cm^{-1}: 831, 1012, 1178, 1232, 1516, 1729. ^{1}H NMR (500 MHz, $CDCl_3$) δ 0.82 (s, 3H), 0.90 (s, 3H), 1.01 - 1.12 (m, 5H), 1.44 - 1.76 (m, 6H), 2.56 - 2.66 (m, 3H), 2.90 (t, *J* = 7.8 Hz, 2H), 3.62 (d, *J* = 13.4 Hz, 1H), 3.67 (s, 3H), 3.79 (d, *J* = 13.4, 1H), 5.02 (s, 2H), 6.90 (d, *J* = 8.5 Hz, 2H), 7.12 (d, *J* = 8.5 Hz, 2H), 7.31 - 7.41 (m, 4H). ^{13}C NMR (126 MHz, $CDCl_3$) : 12.2, 20.5, 20.6, 27.3, 30.1, 36.0, 36.8, 38.7, 45.3, 46.7, 48.4, 51.6, 52.2, 66.1, 69.9, 114.8 (2C), 127.5 (2C), 128.3 (2C), 129.2 (2C), 132.8, 135.4, 141.1, 157.3, 173.4. HRMS for $C_{28}H_{37}O_3N_1^+$ calcd 435.2768, found 435.2760 $[M]^+$.

3-(4-{[4-({[(1R,2R,4R)-1,7,7-trimethylbicyclo[2.2.1]heptan-2-yl]amino}-methyl)phenyl]methoxy}phenyl)propanoic acid hydrochloride QS-528

To an ice-cooled solution of methyl ester **6** (0.195 mmol) in THF (2 ml) a solution of lithium hydroxide monohydrate (0.390 mmol) in water (2 ml) was added. After completion of the reaction (controlled by TLC) THF was evaporated under reduced pressure. 2M hydrochloric acid was added drop-wise until pH 2-3. Precipitate was filtered and washed with water. White solid, 65% yield. M.p. 153.2°C. $[\alpha]_D^{26}$ -35 (c 0.052, EtOH). IR (KBr,) ν/cm^{-1}: 827, 1244, 1383, 1423, 1512, 1709. ^{1}H NMR (500 MHz, DMSO-d6) δ 0.72 - 0.82 (m 3H), 0.85 - 1.04 (m, 8H), 1.41 - 1.54 (m, 2H), 1.55 - 1.65 (m, 1H), 1.71 (t, *J* = 4.1 Hz, 1H), 2.06 - 2.18 (m, 1H), 2.42 - 2.53 (m, 2H), 2.67 - 2.79 (m, 2H), 2.91 (dd, *J* = 8.5 Hz and *J* = 5.4, 1H), 4.05 - 4.21 (m, 2H), 5.09 (s, 2H), 6.89 (d, *J* = 8.5 Hz, 2H), 7.12 (d, *J* = 8.4 Hz, 2H), 7.47 (d, *J* = 7.9 Hz, 2H), 7.67 (d, *J* = 7.9 Hz, 2H), 8.7 (br.s, 2H) 12.05 (br.s, 1H). ^{13}C NMR (126 MHz, DMSO-d6) δ 11.8 , 19.9, 20.4, 26.3, 29.6, 34.7, 35.7, 36.3, 44.3, 47.0, 48.6, 50.1, 64.2, 68.8, 114.8 (2C), 127.8 (2C), 129.3 (2C), 131.0 (2C), 133.2, 138.2, 156.6, 173.9. HRMS for $C_{27}H_{35}O_3N_1^+$ calcd 421.2615, found 421.2610 $[M]^+$. Anal calcd for $C_{27}H_{36}ClNO_3$: Cl 7.74. Found: Cl 8.06.
